# Refractory Hepatic Hydrothorax: A Rare Complication of Systemic Sclerosis and Presinusoidal Portal Hypertension

**DOI:** 10.1155/2018/2704949

**Published:** 2018-04-30

**Authors:** Gary A. Abrams, Robert Chapman, Samuel R. W. Horton

**Affiliations:** ^1^Gastroenterology & Liver Center, Greenville Health System, University of South Carolina School of Medicine Greenville, Greenville, SC, USA; ^2^Department of Pathology, Greenville Health System, University of South Carolina School of Medicine Greenville, Greenville, SC, USA

## Abstract

We report on a rare case of refractory hepatic hydrothorax in an individual with Scleroderma/CREST syndrome and noncirrhotic portal hypertension. Portal pressure measurements revealed a normal transjugular hepatic venous portal pressure gradient, mild pulmonary hypertension, and an unremarkable liver biopsy except for mild sinusoidal dilation. Pulmonary hypertension, cardiac diastolic dysfunction, and chronic kidney disease were determined to be the causes of his refractory pleural effusions and ascites. Over the year, he underwent 50 thoracenteses and 20 paracenteses averaging 10–12 liters/week. Repeat pulmonary evaluation determined his pulmonary pressures to be normal and a secondary review of the “unremarkable” liver biopsy noted mild venous outflow obstruction and possibly Nodular Regenerative Hyperplasia (NRH). Repeat portal pressures indirectly and directly confirmed the existence of presinusoidal portal hypertension that has been associated with NRH. A transjugular intrahepatic portal systemic shunt (TIPS) was placed and he has not required thoracentesis or paracentesis over the past 18 months.

## 1. Introduction

Idiopathic noncirrhotic portal hypertension (INCPH) has many etiologies, but a common denominator is vascular resistance at various locations that include the intrahepatic sinusoidal and presinusoidal as well as extrahepatic portal and hepatic veins [1]. Schistosomiasis is a common worldwide illness and the most frequent cause of INCPH [2]. Nodular Regenerative Hyperplasia (NRH) was first described in 1959 [3]; however this is a histological diagnosis that can often be overlooked. We describe a complicated rare case of refractory right-sided pleural effusions and ascites due to NRH and presinusoidal portal hypertension that was successfully treated with a transjugular intrahepatic portal systemic shunt (TIPS).

## 2. Case Report

A 59-year-old Caucasian male was referred to our liver center for refractory right-sided pleural effusion and abdominal ascites. His history is significant for Scleroderma/CREST syndrome and chronic kidney disease (CKD). He had 12 paracenteses in 2015 and starting from February 2016 was undergoing thoracentesis 3 times weekly (about 8-9 liters/week) and a single weekly paracentesis up to 5 liters. Medications included Spironolactone 50 mg and Furosemide 20 mg, which were limited dosages due to CKD. In February 2016, prior to our visit, he underwent a liver transplant evaluation: Na 138 mg/dL, Cr 4.4 mg/dL, eGFR 31 mL/min, INR 1.0, Hb 13 g/dL, Platelets 342 Th/mm^3^, TB 0.5 g/dL, AlkPhos 278 IU/L, ALT/AST 46/40 IU/L, albumin 2.7 mg/dL, negative viral serology, ANA 1 : 320, SMA and AMA negative, C282Y/H63D, and MELD-Na score 14. Abdominal ultrasound revealed a heterogeneous liver and ascites. A thoracentesis demonstrated a SAAG 1.9 and total protein 3.3 gm/dL suggesting posthepatic portal hypertension. A right heart catheterization was notable for RA 5 mmHg, PA 31/15 mm/Hg, mean 22 mmHg, normal Echo LV function, and grade 1 diastolic dysfunction. At this juncture, the etiology of his presumed cirrhosis had not been determined and a liver biopsy with portal pressures was to be scheduled but he wanted a second opinion and presented to us in March.

**Figure 1:**
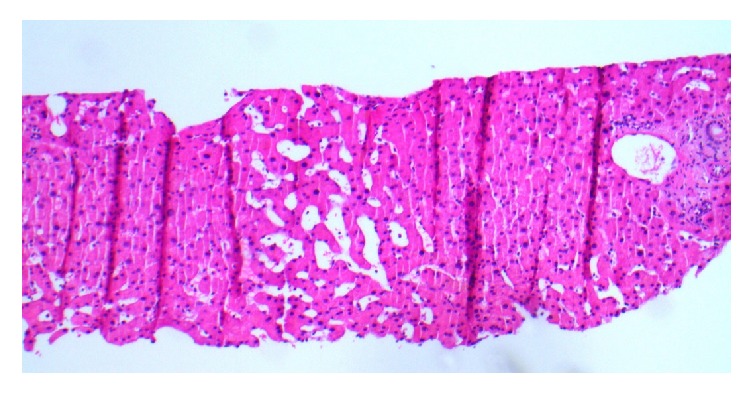
(H&E, 100x) needle core biopsy of hepatic parenchyma demonstrating central vein and sinusoidal dilatation.

**Figure 2:**
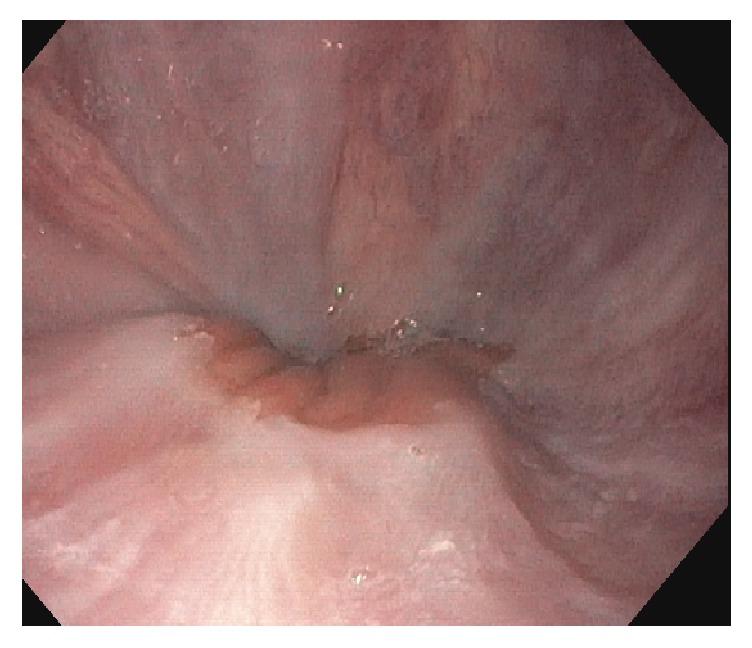
Endoscopic view of a trace varix.

**Figure 3:**
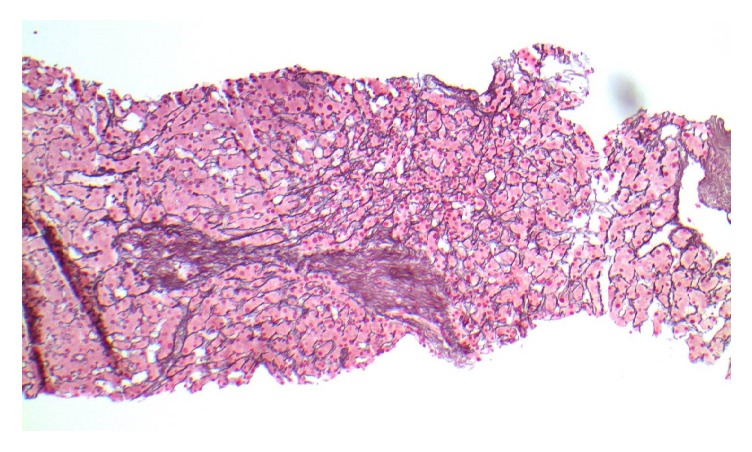
(Reticulin, 100x) hepatic parenchyma exhibiting subtle nodularity without significant fibrosis.

**Figure 4:**
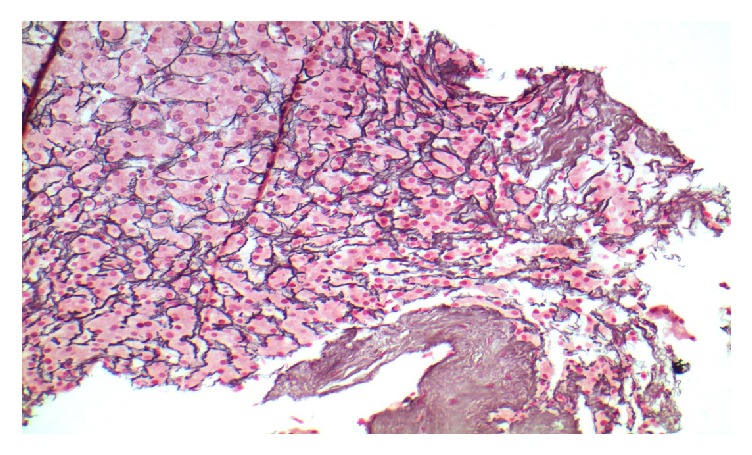
(Reticulin, 200x) hepatic plate thinning and compression adjacent to an area of nodularity.

The physical exam revealed a pleasant frail appearing gentleman with stable vital signs: B/P 87/58, HR 80, and BMI 24.5 kg/m^2^; labs demonstrated TB 0.5 md/dL, AlkPhos 462 IU/L, ALT/AST 62/65 IU/L, total protein 5.7 IU/L, albumin 3.2 mg/dL, and Cr 2.4 mg/dL (spironolactone had been discontinued one month earlier). A large right-sided-pleural effusion with moderate abdominal ascites was noted on examination. He underwent a transjugular intrahepatic portal systemic shunt study (TIPS) and liver biopsy after 100 gm of IV albumin (given for renal dysfunction) with the following results: RA 13 mmHg, FHVP 16 mmHg, WHVP 17 mmHg, and HVPG 1 mmHg (Table 1). The TIPS was aborted due to the normal sinusoidal portal pressure gradient and elevated right-sided pressures. A right heart catheterization 5 hours later revealed RA 13 mm/Hg, PA 57/30 mm/Hg, mean 39 mm/Hg, PWP 20, and C.O 5.5 L/min. Presumptive diagnosis was mild/moderate mixed arterial and venous pulmonary hypertension. The liver biopsy revealed mild sinusoidal dilatation, no inflammation or fibrosis, trace iron deposition and was considered unremarkable, other than mild outflow obstruction (Figure 1). The patient was subsequently referred for a pulmonary work-up as well as a dysphagia evaluation. Pulmonary function tests demonstrated a low DLco 9.33 L, vital capacity 2.75 L, and a repeat heart catheterization after a thoracentesis and paracentesis without albumin: RA 3 mm/Hg, PA 30/10 mm/Hg, mean 18 mm/Hg, and PWP 12 mm/Hg (Table 1). An EGD demonstrated trace esophageal varices (Figure 2). A second interpretation of the liver biopsy noted mild venous outflow obstruction and possibly Nodular Regenerative Hyperplasia (NRH, Figures 3 and 4). Taken together, in the setting of normal right heart pressures and a possible diagnosis of NRH, the patient could have portal hypertension due to a presinusoidal obstruction. He underwent a repeat shunt study this time with direct and indirect portal pressures: RA 2 mm/Hg, FHVP 4 mm/Hg, WHVP 5 mm/Hg, PVP 15 mm/Hg, and portal pressure gradient (PPG = PVP minus FHVP) 11 mm/Hg (Table 1). He successfully underwent a TIPS shunt with a post-TIPS PPG of 7 mm/Hg. Prior to TIPS, the patient underwent 50 thoracenteses and 20 paracenteses over that past year and after TIPS he had 1 thoracentesis/paracentesis 10 days after and none over the past 18 months. Other than mild hepatic encephalopathy, controlled on medication, no other adverse effects have been reported.

## 3. Discussion

Idiopathic noncirrhotic portal hypertension (INCPH) has been proposed to unify the obliterated vasculopathy that links various etiologies [4]. Five diagnostic criteria must be met to diagnose INCPH: (1) clinical evidence of portal hypertension, (2) absence of cirrhosis or advanced fibrosis on liver biopsy, (3) intrahepatic etiologies of liver disease such as viral hepatitis and fatty liver disease, (4) Sarcoidosis, Schistosomiasis, and congenital hepatic fibrosis, and (5) patent portal and hepatic veins [4]. Nodular Regenerative Hyperplasia (NRH) is one subtype of INCPH histologically characterized by liver nodularity, hyperplasia, and no fibrosis [5]. NRH has been associated with many rheumatologic and vascular disorders [6] suggesting a myriad of autoimmune, inflammatory, or neoplastic mechanisms of injury. To date, only 22 cases of Systemic Sclerosis/CREST associated with NRH have been published in a recent systematic review [7].

An initial assessment of ascites and pleural effusions includes a simple diagnostic paracentesis testing the serum-albumin-ascites-gradient (SAAG) and total protein content. In our subject, the SAAG was 1.9 consistent with portal hypertension, and together with a total protein of 3.1 mg/dL both are suggestive for a posthepatic etiology of the ascites [8]. Pulmonary hypertension has been commonly reported in subjects with Systemic Sclerosis [9] and this led to the subject's pulmonary and cardiac evaluation. Why were the posthepatic portal and right heart catheterization pressures on the same day spuriously elevated? We speculate that the albumin infusion immediately prior to these measurements temporarily increased the vascular pressures. Albumin infusions are used prior to a large volume paracentesis to prevent postparacentesis circulatory renal dysfunction especially in subjects with baseline renal impairment [10]. We have recently become aware of two other individuals, in our practice, which had elevated right-sided portal pressures after albumin infusions that were normal without a prior colloid transfusion. Hence, we have altered our practice whereby patients scheduled for portal pressure measurements for refractory ascites warranting prophylactic albumin infusions will undergo a large volume paracentesis several days prior to pressure assessments.

Portal pressure measurements are the primary diagnostic approach to establish the anatomic pathogenesis of portal hypertension [11]. The FHVP should be within a couple of mmHg higher than the RA pressure. The WHVP indirectly estimates the portal pressure and if the difference between the two measurements, called the HVPG, is greater than 6 mmHg, this signifies sinusoidal portal hypertension. Postsinusoidal portal hypertension is defined by an elevated FHVP and WHVP but a normal HVPG. In comparison, presinusoidal portal hypertension cannot be diagnosed by these indirect portal pressure measurements alone, requiring direct calculation of the portal venous system. A clinically significant HVGP is considered ≥ 10 mmHg and predicts the development of ascites or esophageal varices and clinical decompensation in subjects with compensated cirrhosis [12].

The patient's initial portal pressures were consistent with posthepatic portal hypertension. The liver biopsy did not reveal cirrhosis; therefore pulmonary hypertension and mild diastolic dysfunction were determined to be the culprits of the pleural effusions and ascites. However, a subsequent pulmonary evaluation revealed mild pulmonary dysfunction and normal right heart pressures. During this period he had an EGD for dysphagia, and trace esophageal varices were noted. Finally, a repeat interpretation of the liver biopsy suggested NRH. Taking these together, we were suspicious of a presinusoidal etiology for his effusions/ascites and directly measured the portal vein along with indirect measurements. The portal pressure was 15 mmHg and the PPG was 11 mmHg, consistent with portal hypertension; therefore we proceeded with TIPS placement. Ten days after TIPS he needed a single 3-liter thoracentesis and subsequently has not warranted any further thoracentesis or paracentesis over the past 1.5 years. A recent review investigated the outcome of TIPS in 25 subjects with NCPH [13]. NRH was identified in 12 subjects and associated with Scleroderma in only 1 person in this study. Eight of nine NRH individuals had a normal WHVP; all 12 had elevated portal pressures and 9/10 had an elevated HVPG. Indications for TIPS included either esophageal varices or ascites; none of the NRH subjects had hepatic hydrothorax listed as an indication. The long-term outcome over 3 years was very good with 80% functioning TIPS and hepatic encephalopathy was the most common adverse effect.

An international working definition of liver nodules defined NRH in 1995 [14]. It is postulated that the underlying pathogenesis for presinusoidal portal hypertension is obliteration of the small portal venules [15] and abnormal electron dense deposits within the hepatic microcirculation [16]. The immunologic or immunogenetic risk factors for these microvasculopathies are unknown. The association of NRH and Scleroderma has been limited to case reports but the recent systemic review does suggest common clinical manifestations including Raynaud's phenomenon in 19/19 individuals (as did our case), ascites in 6/8, and varices in 10/13 of individuals [7]. Pulmonary information was limited to only 9 subjects with dyspnea; however the exact etiology is unclear and pulmonary hypertension or fibrosis was mentioned. To our knowledge, there are no reported cases of refractory hepatic hydrothorax in the literature as a complication of Systemic Sclerosis and NRH.

In conclusion, this case highlights an unusual presentation of refractory hepatic hydrothorax and ascites due to the combination of Systemic Sclerosis/CREST syndrome causing NRH and presinusoidal portal hypertension that was successfully treated with a TIPS shunt. This case also brings forth the diagnostic awareness that both indirect and direct portal vein measurements are warranted to diagnose presinusoidal portal hypertension.

## Figures and Tables

**Table 1:** Cardiac and portal pressure measurements.

*Right cardiac catherization (mmHg)*	
Initial	RA 5, PA 31/15 (mean 22)
After albumin	RA 13, PA 57/30 (mean 39), PWP 20, CO 5.5 L/min
PFTs	DLco 9.33 L, VC 2.75 L
*Portal pressure measurements (mmHg)*	
Initial after albumin	FHVP 16, WHVP 17, HVPG 1
Pre-TIPS without albumin	FHVP 2, WHVP 4, HVPG 5, PVP 15, PPG 11
Post-TIPS	FHVP 5, WHVP 7, HVPG 2, PVP 14, PPG 7

RA: right atrial pressure, PWP: pulmonary wedge pressure, CO: cardiac output, FHVP: free hepatic vein pressure, WHVP: wedge hepatic vein pressure, HVPG: hepatic vein pressure gradient, PVP: direct portal venous pressure measurement, PPG: portal pressure gradient (PVP minus WHVP), PFTs: pulmonary function tests, DLco: diffusion capacity of carbon dioxide, VC: vital capacity, TIPS: transjugular intrahepatic portosystemic shunt.
